# Energy supply during nocturnal endurance flight of migrant birds: effect of energy stores and flight behaviour

**DOI:** 10.1186/s40462-024-00479-5

**Published:** 2024-05-30

**Authors:** Susanne Jenni-Eiermann, Felix Liechti, Martins Briedis, Yann Rime, Lukas Jenni

**Affiliations:** Swiss Ornithological Institute, Seerose 1, Sempach, CH-6204 Switzerland

**Keywords:** Bird migration, Endurance flight, Migratory flight behaviour, Multi-sensor data loggers, Physiology of flight, Plasma metabolites, Radar tracks, Energy saving

## Abstract

**Background:**

Migrating birds fly non-stop for hours or even for days. They rely mainly on fat as fuel complemented by a certain amount of protein. Studies on homing pigeons and birds flying in a wind-tunnel suggest that the shares of fat and protein on total energy expenditure vary with flight duration and body fat stores. Also, flight behaviour, such as descending flight, is expected to affect metabolism. However, studies on free flying migrant birds under natural conditions are lacking.

**Methods:**

On a Swiss Alpine pass, we caught three species of nocturnal migrant passerines out of their natural migratory flight. Since most night migrants start soon after dusk, we used time since dusk as a measure of flight duration. We used plasma concentrations of metabolites of the fat, protein, and carbohydrate metabolism as indicators of relative fuel use. We used flight altitudes of birds tracked with radar and with atmospheric pressure loggers to characterize flight behaviour.

**Results:**

The indicators of fat catabolism (triglycerides, very low-density lipoproteins, glycerol) were positively correlated with body energy stores, supporting earlier findings that birds with high fat stores have a higher fat catabolism. As expected, plasma levels of triglycerides, very low-density lipoproteins, glycerol and ß-hydroxy-butyrate increased at the beginning of the night, indicating that nocturnal migrants increased their fat metabolism directly after take-off. Surprisingly, fat catabolism as well as glucose levels decreased in the second half of the night. Data from radar observations showed that the number of birds aloft, their mean height above ground and vertical flight speed decreased after midnight. Together with the findings from atmospheric pressure-loggers put on three species, this shows that nocturnal migrants migrating over continental Europe descend slowly during about 1.5 h before final landfall at night, which results in 11–30% energy savings according to current flight models.

**Conclusions:**

We suggest that this slow descent reduces energy demands to an extent which is noticeable in the plasma concentration of lipid, protein, and carbohydrate metabolites. The slow descent may facilitate the search for a suitable resting habitat and serve to refill glycogen stores needed for foraging and predator escape when landed.

**Supplementary Information:**

The online version contains supplementary material available at 10.1186/s40462-024-00479-5.

## Introduction

Migrating birds are known to fly non-stop for hours and certain species even for days [1–3]. These endurance flights are performed at a high metabolic rate [4]. Moreover, most birds do not feed during migratory flight and must therefore rely on energy and metabolic water derived from body stores. The main energy source are triglycerides (TG) stored in extra-muscular fat deposits which deliver up to 97% of the energy (e.g. [2, 5, 6]). TG in fat deposits are hydrolysed into glycerol (GLYC) and free fatty acids (FFA) and then released into the blood. To meet the high energy demands during flight, migrants have evolved special adaptations to optimize transport and oxidation of FFA [7, 8]. Roughly, these are (a) the transport of the insoluble FFA in the blood by resynthesis of FFA into TG in the liver which are then transported as very low density lipoproteins (VLDL) to the flight muscles [8, 9]; (b) the transport of fatty acids (FA) into the muscle cells by an increase of fatty acid transport proteins [7] and (c) the oxidation of the FA by an upregulation of oxidative enzyme activity [10–12]. However, FA cannot meet all metabolic needs. Energy from lipid stores is not readily available in the high amounts needed at the beginning of flight and FA do not provide glucogenic precursors which are necessary to fill up the intermediates of the citric acid cycle for FA oxidation. Therefore, FA must be complemented by two other fuels, glucose (GLU) and protein. Because glycogen stores are small and partially conserved [13, 14], they do not play an important role. Instead glucogenic amino acids are the main source for glucogenic precursors.

The share of FA and protein to total energy needs changes at the beginning of a long flight and varies with body fat stores. At the beginning of an endurance flight, energy is mainly derived from the muscular and hepatic carbohydrate stores, a transient phase of about 30 to 60 min which progressively leads into a predominantly lipid-based metabolism [15–19]. Protein as a fuel has an 8 times lower energy content per fresh weight than fat, hence is costly to transport and should be minimized [6]. Studies on inactive fasting birds and mammals and three bird species during flight have shown that the relative contribution of protein to energy expenditure decreases with increasing initial fat stores, and reaches a lower limit of around 5% [6], but increases when fat stores are near depletion. However, in wind-tunnel studies protein use during fasting seems to be independent of fat utilisation (and metabolic rate) and mainly driven by tissue-specific turnover and the need to liberate water in dry conditions [2, 20].

Birds during migration vary flight altitude repeatedly to find optimal wind conditions [21, 22] and must descend to land for stopover. Because of these repeated ascents and descents energy expenditure during migratory flight is not constant. The effect of varying energy expenditure on flight metabolism has not been investigated so far in migrating birds.

The investigation of the metabolism during natural endurance flight is technically demanding. Studies about changes of fuel composition during endurance flight have only been done in a wind-tunnel or with homing pigeons after flights of varying length [2, 15, 17, 18, 23–25] and may not be representative of free-flying birds during actual migration, e.g. regarding energy expenditure, water loss and metabolism [4, 21, 26, 27].

The aim of this study, therefore, was to investigate changes in energy supply during the course of long flights and its dependence on energy stores and the flight behaviour in free-living passerines under natural conditions.

At our study site, a Swiss Alpine pass, we were able to catch nocturnal migrant passerines out of their natural migratory flights. Based on the assumption that time elapsed since dusk corresponds approximately to flight time (for evidence see Methods), we investigated changes in energy supply during the night by measuring plasma metabolites of the fat, protein, and carbohydrate metabolism in a cross-sectional study. Plasma metabolite concentrations have been used as indicators of relative fuel use [23, 28, 29], although they cannot generally be equated with metabolite turnover (e.g. [28, 30]; see Discussion). As indicators of lipid catabolism, we measured plasma FFA and GLYC, the products of TG hydrolyzation, and plasma TG and VLDL which indicate the resynthesis of FFA into VLDL in fasting and flying migrants [9, 30]. We also measured the ketone body ß-hydroxy-butyrate (ßOHB), an indicator of the fasting metabolism which is synthesized from FFA and preferentially oxidized in extrahepatic tissues. By inhibiting glucose oxidation, ßOHB and FFA contribute to a glucose-sparing effect [31, 32]. As an indicator of protein catabolism, we measured uric acid (UA), the end-product of nitrogen metabolism [33–35]. Finally, we measured GLU, although it plays a minor role as energy source during long-term exercise and is known to be maintained within narrow limits in the blood [25, 36]. We tested whether plasma metabolite levels changed during nocturnal flight (with time of capture) and to which extent they depended on body energy stores.

We expected an increase of fat catabolism and a reduction of protein degradation in the first hours after take-off which then level off and remain stable for the rest of the flight until the descent for landing. We also expected a positive relationship between energy stores and fat catabolites and a negative relationship with UA, because high initial fat stores have been shown to reduce the relative contribution of protein to energy expenditure and lead to a lipid-based metabolism [6]. We explored whether changes in flight behaviour, notably the final descent to land, changes flight metabolism. We used radar data from two nearby sites and atmospheric air pressure measurements from data loggers put on three species to evaluate variation in flight altitude during the night.

## Materials and methods

### Animals

At the Alpine pass Col de Bretolet (1923 m a.s.l.), Switzerland, free-living birds were caught in 9 m high mist nets out of their nocturnal migratory flight during the autumn migration periods from August to October 1986–1988, 1991, 1992, and 1994. For this study we investigated three species of night-migrating passerines: the two long-distance migrants garden warbler *Sylvia borin* and pied flycatcher *Ficedula hypoleuca*, and the short-distance migrant European robin *Erithacus rubecula* [37].

For each bird, the visible subcutaneous fat deposit in the tracheal pit and on the abdomen was scored (five scores scale, a precursor of the Kaiser 1993 score [38]). The birds were ringed, weighed to 0.1 g, the length of their third-outermost primary was measured [39], and they were thereafter released. Only birds that had finished their post-juvenile or post-breeding moult, and hence were in a migratory state were selected [40].

### Blood sampling and metabolite determinations

Blood was obtained within 3–15 min after the bird flew into the mist net by puncturing the alar vein and collected with a capillary system (Microvette® C8300 Fluore, Sarstedt). The blood was centrifuged within 30 min, and the plasma stored in liquid nitrogen in the field and later at -20° C until analysis in the same year. The measurement of plasma concentrations of the metabolites free fatty acids, glycerol, triglycerides, ß-hydroxy-butyrate, glucose, uric acid followed the procedures described earlier [16]. Lipoprotein levels were determined with the standard agarose gel electrophoresis system Paragon (Beckman), used according to the instructions given by the manufacturer. The lipoproteins were visualised with Sudan Black B and quantified by densitometric scanning (Appraise Junior densitometer, Beckman). The peaks had been characterized previously by ultracentrifugation [9]. The fraction (percentage) of very-low-density lipoproteins (VLDL) was used for this study. Because the amounts of collected blood varied, not all metabolites could be determined in all individuals. Metabolites are given in mmol/l and VLDL as a percentage of fraction 1 of the electrophoresis.

### Energy stores

For each individual, we calculated a measure of energy stores which was the percentage of body mass (BM) above lean mass (LM): (BM-LM)/LM*100. Birds without visible subcutaneous fat stores were considered to be at lean mass. We used all available individuals caught at Col de Bretolet without visible subcutaneous fat stores between 1988 and 2020 (*n* = 5328 European robins, 744 pied flycatchers, 161 garden warblers) to establish a relationship between lean body mass and size (third-primary length) for each species. From these relationships, we calculated LM for each individual according to its size, and the difference to actual body mass (BM-LM).

Some of the birds investigated were below lean mass. However, birds at LM (0% energy stores), still comprise appreciable amounts of energy reserves because (a) of remaining invisible fat stores and (b) because energy can still be derived by catabolizing muscles and other organs. Birds with 0% energy stores in our case therefore are well above structural mass, the mass of live birds shortly before starvation death which show both no visible subcutaneous fat deposits and emaciated breast muscles (fat and muscle score both zero; [41]). For the garden warbler our birds with 0% energy stores have a mean body mass of 16.78 g, while birds at structural mass weigh 12.8 g [41]; for pied flycatchers, mean body mass at 0% energy stores is 11.45 g versus 7.8–9 g structural mass (original data used in [42]; no structural mass data available for European robin). Energy stores well above LM consist mainly of fat, while a major part of the energy reserves below LM is protein [42, 43]. Protein has an 8 times lower energy content per fresh weight than fat [6].

### Start and duration of nocturnal migration

According to radar data from southern Germany and Switzerland, i.e., on the continent without interfering sea, the majority of nocturnal migrants in autumn start their migratory flights within about an hour after civil twilight (which is 0.5–1 h after sunset, depending on season). Numbers aloft decrease during the second part of the night, indicating that many night migrants land during the night [44–47]. This pattern is confirmed for the study site Col de Bretolet (see Additional file). Soon after twilight, migration traffic rates increase and peak around midnight. This indicates that many birds have their stopover sites not in the Alps but further north, hence they need some time to arrive at the Alpine pass. Before morning twilight, all night migrants have landed (no day captures of night migrants in the high mist nets on Col de Bretolet).

A start of nocturnal migration within 69 min after civil twilight was also observed in 400 tracked individuals of nine North American passerine species, while non-migratory regional movements may start throughout the night [48]. Generally, this also holds for European passerines (e.g. [49, 50]). Non-migratory regional movements may also pertain to birds observed to start movements later during the night in northern Europe, but some birds may start true migration only later during the night depending on energy stores and weather conditions [50–59]. From these findings, we assumed for our study site in a simplified way that nocturnal migrants generally start soon (within about an hour) after sunset and land during the night or before dawn at the latest. Hence, we assumed that the time between evening civil twilight and capture at the Alpine pass is an approximate measure of flight duration. Because we were also interested in the changes in metabolites in the few hours before civil dawn, when all night migrants flying over continental Europe land, we expressed flight duration as the percentage of nighttime elapsed between evening twilight and capture, rather than hours after dusk, and so accounted for the variable night-length between August and October. Analysis of the data with flight duration (in h) after dusk yielded very similar results (not shown).

### Analysis of metabolite data

To estimate the effects of energy stores and flight duration on metabolite levels, we used general linear models with the metabolite or VLDL as the dependent variable, and species, time span between capture and end of blood sampling (time after capture; min), energy stores (percentage), and flight duration (percentage of nighttime elapsed between evening twilight and capture) as independent variables. To account for possible non-linear relationships, we also included energy stores squared, flight duration squared and time after capture squared. To consider possible different relationships between species, we included the interaction terms of all these variables with species in the initial model. The interactions and quadratic terms were removed stepwise backwards if they did not reach significance at the *P* < 0.05 level.

### Radar data

Two types of data were collected from radars: the distribution of migrants across altitude and flight tracks of single migrants [60, 61].

In 2007, a mobile pencil beam x-band (simplified “Superfledermaus”) radar, specifically adapted to detect birds [62, 63], was operated at Planachaux, 4.5 km northeast of Col de Bretolet on a plateau at the northern flank of the main valley at 1677 m a.s.l. from 6 August until 29 October 2007 (see [64] for details and a map). The radar beam was directed perpendicularly to the direction of the main valley (which is the main migratory direction) and recorded migrating birds flying towards Col de Bretolet at elevations from 300 m below the radar in the valley up to 5300 m above the radar every half hour (for details see [64]). About 80% of bird echoes are from small passerines [65]. Passerines migrating at night generally fly solitary or with distances between individuals large enough to be recognized as individuals by the radar [66]. From the number of bird echoes per height interval of 50 m and hour, we calculated the migration traffic rate (MTR) per height interval and hour which is the number of birds crossing a line of 1 km perpendicular to the migratory direction for one hour (birds km^− 1^ hour^− 1^). To account for the seasonal shift in dawn and dusk twilight (51–59 min within a month), we defined dusk to occur in the hour 20:00–20:59 (UTC-1) in August, 19:00–19:59 in September and 18:00–18:59 in October. The corresponding dawn hours were at 4:00–4:59 in August, 5:00–5:59 in September and 6:00–6:59 in October. Total sample size of radar bird echoes was 88,653. We calculated the mean height of the migrating birds (± SD) for the four hours at and after dusk and for the 6 h before and during dawn, while the data of the remaining hours in the middle of the night (2 h in September and 4 h in October) were allocated to the middle of the night.

In 1988, a tracking radar Superfledermaus [63] was set up at Col de la Croix (1718 m a.s.l.) from 1 August – 9 October, an Alpine pass 32 km northeast of Col de Bretolet with similar topographic characteristics. In a radius of about 4 km, solitary night migrants were tracked and their flight paths recorded for up to about 4 min. The type of bird (small and large passerines, small and large waders, swifts) was determined from the wing-beat pattern (see [67–69]). We excluded birds with reverse flight directions (180° around NE) and used all 2972 flight paths of birds classified as small passerines.

The flight behaviour of migrants differs between tail- and headwind conditions. With headwinds, birds fly lower above ground and follow the valleys to avoid strong headwinds, hence descend when having crossed an Alpine pass. With tailwinds, birds fly higher and are less affected by topography [70–72]. Therefore, we analysed vertical speed separately for tail- and headwind conditions. As above, we related mean vertical speed to the hours relative to dusk and dawn.

### Logger data

We used data from three night-migrant species – great reed warbler *Acrocephalus arundinaceus*, Eurasian hoopoe *Upupa epops*, and northern wheatear *Oenanthe Oenanthe* – equipped with multi-sensor data loggers (geolocators; model GDL3-PAM, Swiss Ornithological Institute) recording body acceleration, ambient light, and atmospheric pressure [73]. Accelerometer data were recorded every 5 min and used to identify the beginning and end of migratory flights, whereas light data were used to distinguish between nocturnal and diurnal flights [74, 75]. Atmospheric pressure was recorded every 30 min and converted in elevation above sea level using International Standard Atmosphere model [76]. Due to its smaller size, loggers of northern wheatears had no accelerometers, hence the timing of the end of migratory flights had an accuracy of only 30 min [49].

We analysed flight heights during the first flight bouts of autumn migration over continental Europe of at least 2 h duration. Great reed warblers were tracked between 2015 and 2017 and started in Bulgaria, Czech Republic, and Russia (Kaliningrad Oblast) [77], Hoopoes were tracked between 2015 and 2016 in the Valais in Switzerland [78], and northern wheatears were tracked between 2016 and 2020 and started in southern Switzerland [49]. We used 89 flights of 34 individuals great reed warblers [79], 49 flights of 20 Eurasian hoopoes [80], and 11 flights of 7 northern wheatears [81].

#### Flight simulation

To estimate the energy savings of descending flight compared to horizontal flight, we calculated mechanical power for maximum range speed across the observed range of sinking speeds. Since only flight angles can be entered in the simulation software, we first calculated the maximum range speed over a range of flight angles (0–10°) and then calculated the sinking speed based on the resulting maximum range speed. Finally, we selected the range covering the observed sink speeds (0 – -0.3 m/s corresponding to 0–2.2°). We used the R-package afpt ([82], based on [83]) with species specific morphometrics (body mass, wing span, wing area) taken from Bruderer et al. [84].

## Results

### Effect of energy stores and flight duration on metabolite concentrations

**Fig. 1:**
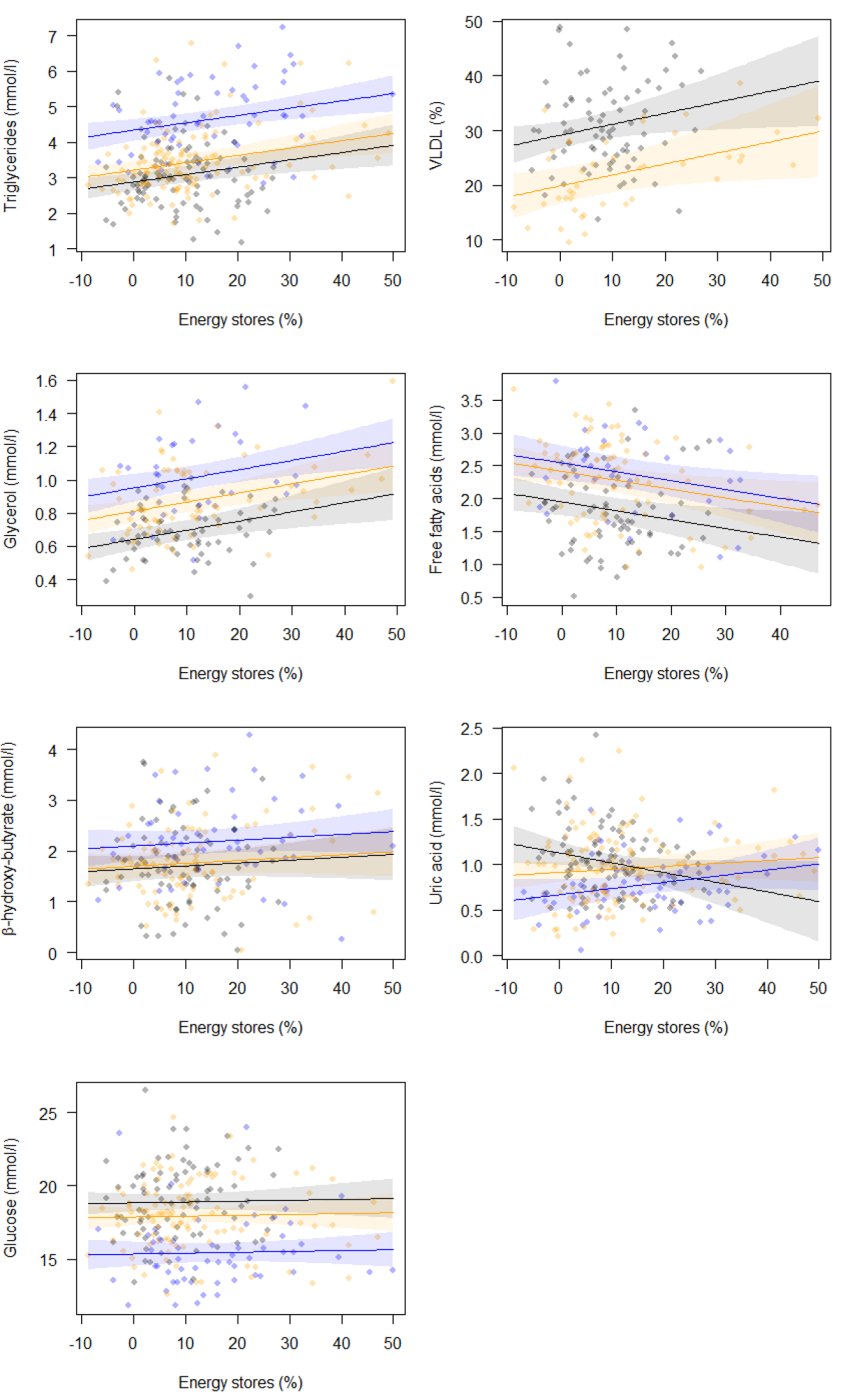
Dependence of plasma concentrations of six metabolites and the proportion of VLDL on body energy stores (percent above lean mass, see Methods). The lines (with 95% confidence intervals) are linear relationships derived from the general linear models presented in Table 1. Dots are raw data corrected for time after capture and flight duration (nighttime). Grey dots and lines are for European robins, orange for pied flycatchers and blue for garden warblers

TG, VLDL and GLYC were significantly positively related with body energy stores, while FFA was negatively related. UA was negatively related with body energy stores in the European robin, but not in the two other species. ßOHB and GLU showed no significant correlation with energy stores (Table 1; Fig. 1).

**Table 1:** General Linear Models with one of the six metabolites (mmol/l) or VLDL as response variable, species as factor (European Robin ER, Pied Flycatcher PF, Garden Warbler GW), and body energy stores (percent above lean mass, see Methods), flight duration (percentage of nighttime elapsed between evening twilight and capture, see Methods), (body energy stores)^2^, (flight duration)^2^, time elapsed between capture and blood sampling (time after capture; min), (time after capture)^2^ and interactions with species as fixed factors. Interactions and quadratic terms were removed stepwise backwards if they did not reach significance at the *P* < 0.05 level. Sample size per species (in parentheses), estimates ± SE and significance (* *p* < 0.05, ** *p* < 0.01, *** *p* < 0.001) are given

		Triglycerides		ß-Hydroxy-butyrate		Free fatty acids		Glycerol		Uric acid		Glucose		VLDL	
		(n)	B ± SE	(n)	B ± SE	(n)	B ± SE	(n)	B ± SE	(n)	B ± SE	(n)	B ± SE	(n)	B ± SE
Intercept			3.863 ± 0.387***		2.912 ± 0.394***		1.439 ± 0.264***		0.558 ± 0.104***		0.794 ± 0.116***		17.379 ± 0.731***		4.217 ± 4.557
Species	ER	(92)	-1.453 ± 0.165***	(78)	-0.451 ± 0.152**	(69)	-0.598 ± 0.132***	(62)	-0.310 ± 0.045***	(95)	0.463 ± 0.106***	(83)	3.492 ± 0.442***	(76)	9.383 ± 1.553***
	PF	(119)	-1.122 ± 0.158***	(89)	-0.400 ± 0.147**	(79)	-0.134 ± 0.129	(71)	-0.141 ± 0.044**	(117)	0.251 ± 0.098*	(105)	2.524 ± 0.417***	(42)	0
	GW	(58)	0	(51)	0	(31)	0	(32)	0	(56)	0	(57)	0		
Energy stores			0.021 ± 0.006***		0.006 ± 0.006		-0.014 ± 0.005**		0.006 ± 0.002**		0.007 ± 0.004		0.006 ± 0.015		0.201 ± 0.090*
Energy stores*species	ER										-0.017 ± 0.007*				
	PF										-0.003 ± 0.005				
	GW										0				
Flight duration			2.761 ± 1.212*		3.221 ± 1.103**		0.297 ± 0.226		1.458 ± 0.330***		-0.178 ± 0.109		-2.694 ± 0.683***		60.449 ± 15.443***
(Flight duration)^2^			-3.022 ± 1.107**		-3.005 ± 0.997**				-0.973 ± 0.307**		0				-68.539 ± 12.773***
Time after capture			-0.021 ± 0.017		-0.392 ± 0.066***		0.202 ± 0.048***		-0.011 ± 0.004*		0.005 ± 0.007		-0.087 ± 0.049		0.518 ± 0.187**
(Time after capture)^2^					0.22 ± 0.004***		-0.009 ± 0.003**								

**Fig. 2:**
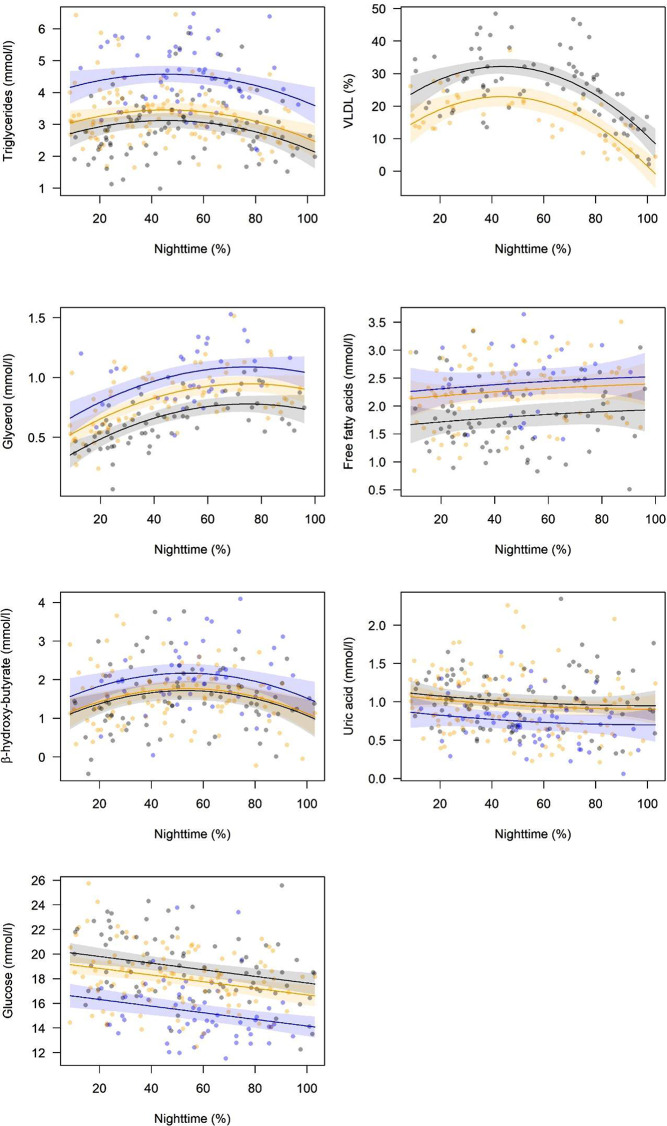
Dependence of plasma concentrations of six metabolites and the proportion of VLDL on nighttime passed (percentage of nighttime elapsed between evening twilight and capture, see Methods). The lines (with 95% confidence intervals) are linear relationships derived from the general linear models presented in Table 1. Dots are raw data corrected for time after capture and body energy stores. Grey dots and lines are for European robins, orange for pied flycatchers and blue for garden warblers

When accounting for energy stores at capture in the models, all metabolites derived from lipids, except FFA, changed significantly with progressing nighttime (Table 1). TG, VLDL, ßOHB and GLYC concentrations showed a curvilinear course: they increased during the first hours of the night and decreased towards dawn. GLU and UA (non-significantly) decreased linearly during the night (Table 1; Fig. 2).

The relationships between metabolites and body energy stores or nighttime were parallel between the three species (interactions with species not significant), except between UA and energy stores. However, the levels differed: garden warblers had the highest levels of all fat metabolites, and the lowest levels of GLUC and UA, while the opposite held for European robins; pied flycatchers were between them.

### Flight behaviour

**Fig. 3:**
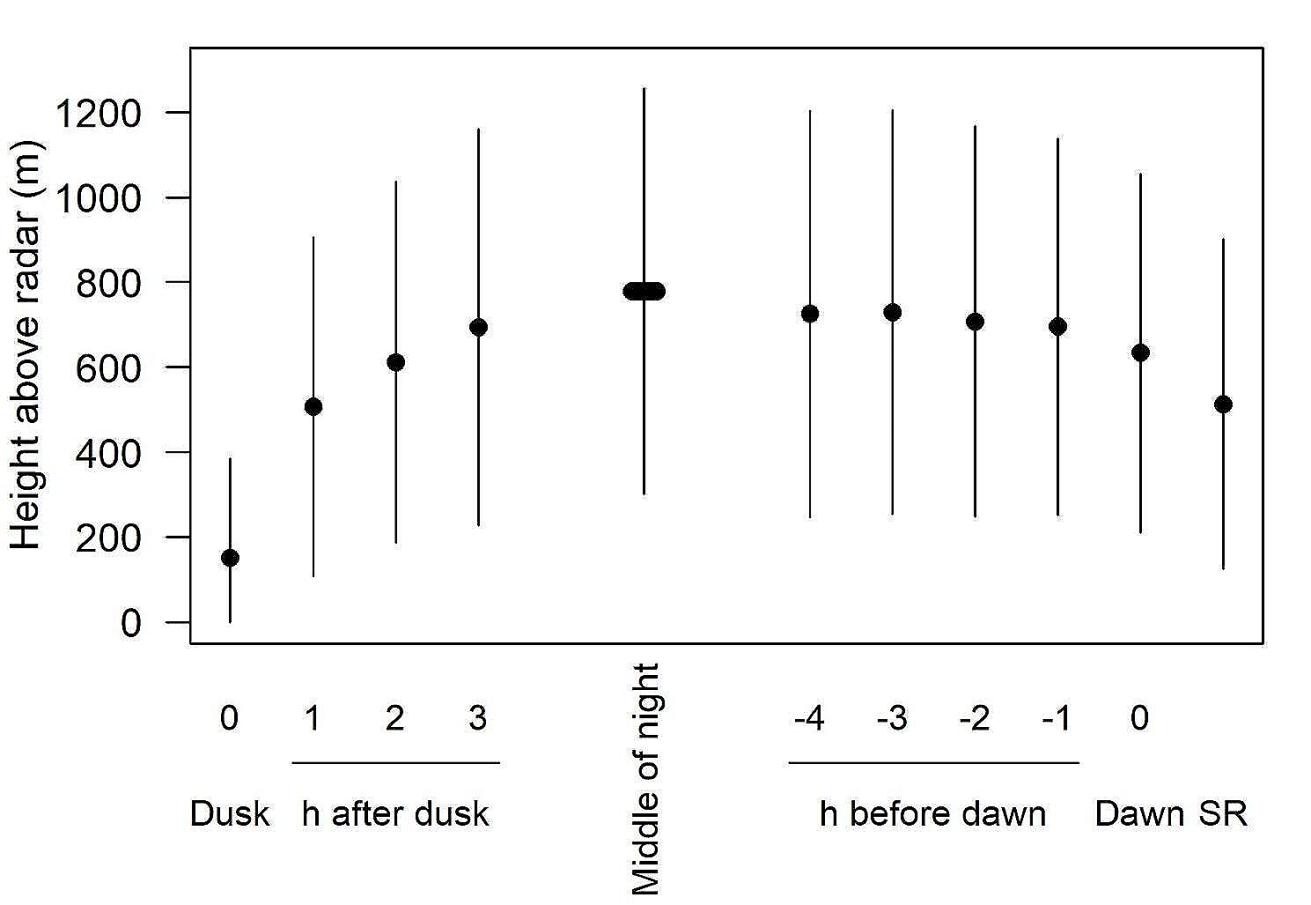
Mean (± SD) height of birds aloft during August – October at the site Planachaux near Col de Bretolet. Data are given for the hour including dusk and the following three hours, for the hours in the middle of the night, for the 4 h before dawn, the hour including dawn and the hour including sunrise (SR)

The mean height of birds above the radar near Col de Bretolet increased steeply after dusk. Birds flew highest in the middle of the night. About 2–3 h before dawn, mean height decreased by about 100 m and decreased more steeply at sunrise (Fig. 3).

**Fig. 4:**
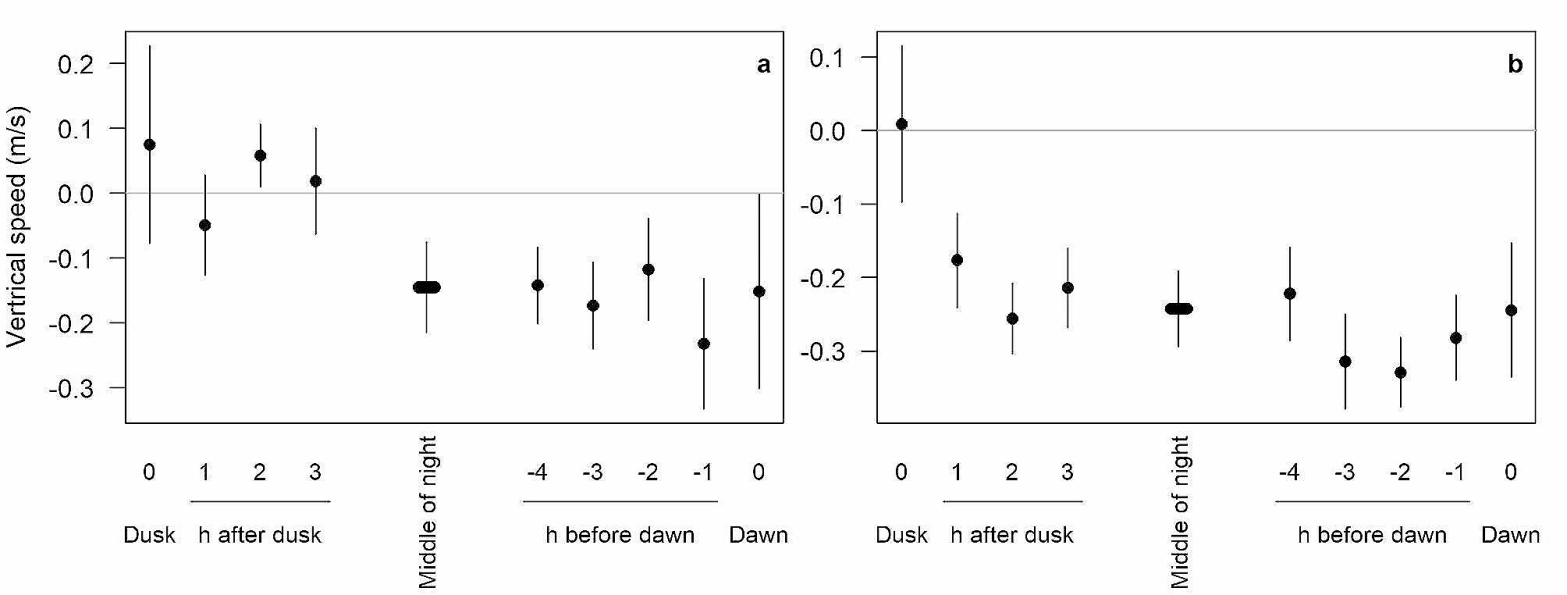
Mean vertical speed (± SE) of small passerines over Col de la Croix under tailwind (**a**) and headwind (**b**) conditions. Data are given for the hour including dusk and the following three hours, for the hours in the middle of the night, for the 4 h before dawn and the hour including dawn

Under tailwind conditions at Col de la Croix, the flight paths of small passerines were horizontal during the first hours of the night. Later, they descended progressively with mean sinking speeds increasing from − 0.14 m/s in the middle of the night to -0.23 m/s one hour before dawn (Fig. 4a). Under headwinds, all birds descended, except during the hour of dusk when night migration started. Migrants are well known to fly lower above ground through valleys under headwinds, hence descend when having crossed the Alpine pass. However, mean sinking speed increased from around − 0.22 m/s to -0.3 m/s in the two hours before dawn (Fig. 4b).

**Fig. 5:**
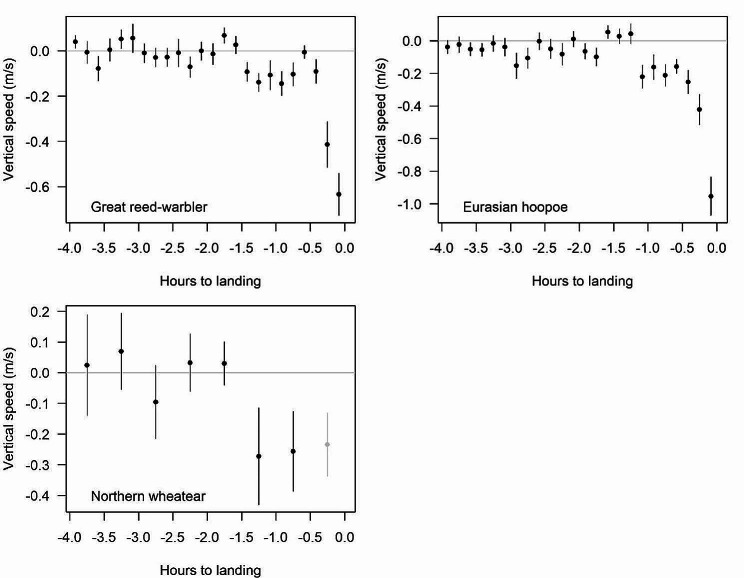
Mean vertical speed (± SE) during the 4 h before landing (at 0 h) for great reed warblers, Eurasian hoopoes and northern wheatears. Because landing time of northern wheatears has an accuracy of only 30 min, the value before landing is unreliable (indicated in grey)

Great reed warblers, Eurasian hoopoes and northern wheatears on average flew horizontal until about 1.5 h before landing. Thereafter, they descended slowly before a final steep descent 10–20 min before landing (Fig. 5). During 90–20 min before landing, mean sinking speed was − 0.091 m/s for great reed warblers, -0.201 m/s for Eurasian hoopoes during 70–20 min before landing, and − 0.265 m/s for northern wheatears during 90–30 min before landing. The data of great reed warblers and Eurasian hoopoes, with an accuracy of landing time of 5 min, indicate that final descent was very steep. In northern wheatears, landing time had an accuracy of only 30 min, hence vertical speed within 30 min before landing is unreliable.

**Fig. 6:**
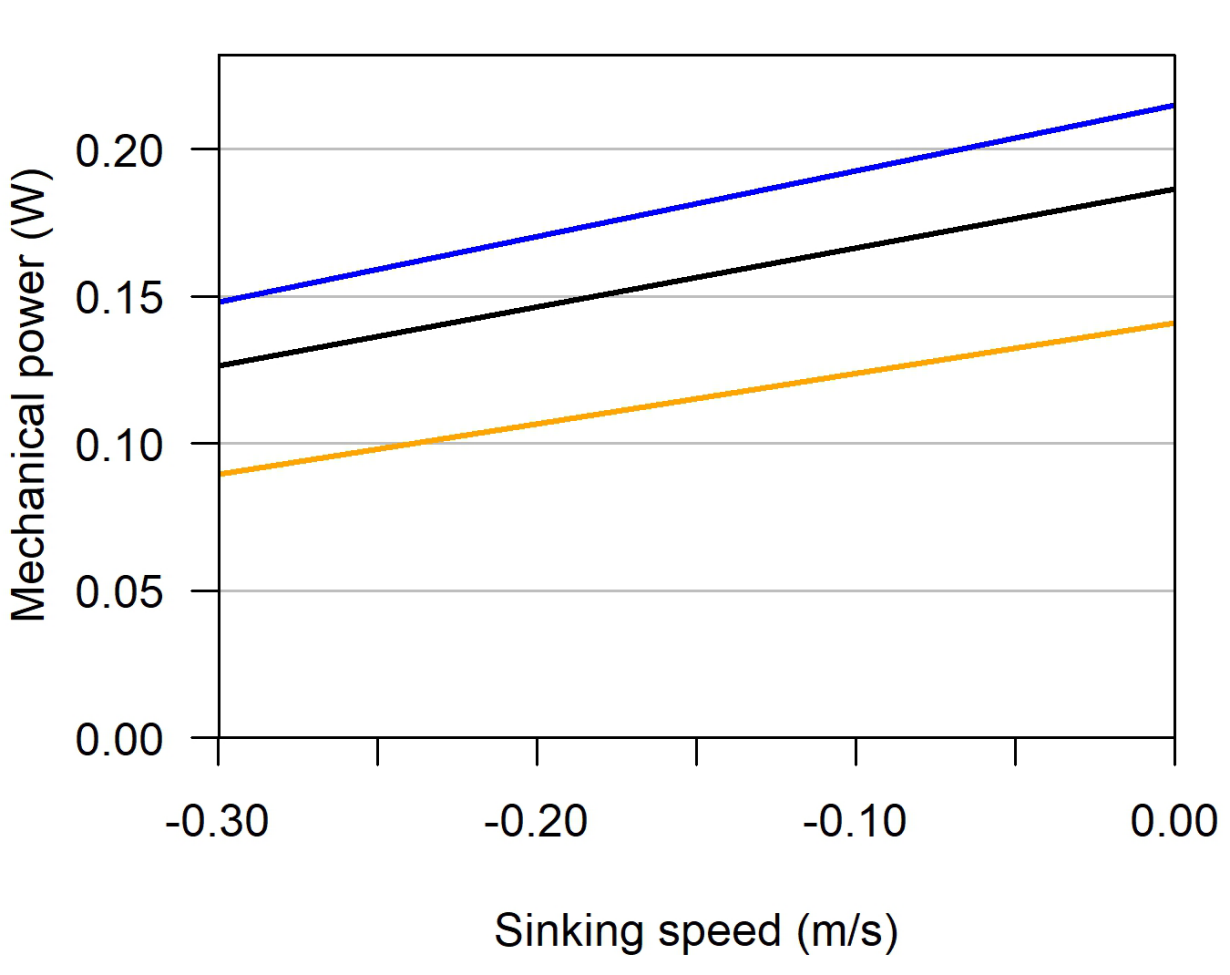
Mechanical power for flight depending on sinking speed for (from top) garden warbler, European robin and pied flycatcher, calculated with the package by Klein-Heerenbrink and Hedenström [82]

The theoretical flight model [82] reveals that sinking speeds of -0.1 – -0.26 m/s, as occurring in the radar tracks and the species followed by atmospheric pressure-loggers before the steep descent, reduce mechanical power of the three species analysed for metabolites by 11–30% (Fig. 6).

## Discussion

The increase at the beginning of the night of the plasma levels of TG, VLDL, GLYC and ßOHB suggests that nocturnal migrants increased their fat metabolism directly after take-off. Fat catabolites peaked in the middle of the night. Surprisingly, thereafter they decreased to a slightly but significantly lower level. In addition, the plasma concentrations of TG, VLDL and GLYC were positively correlated with body energy stores supporting earlier findings that birds with high fat stores have a higher fat catabolism. The radar data showed that the number and mean height above ground of birds aloft, and the vertical flight speed of small passerines decreased after midnight. Together with the findings from the individual birds tracked with atmospheric pressure-loggers, this suggests that nocturnal migrants over continental Europe prepare for landfall over about 1.5 h. Therefore, we conclude that this slow descent before landing reduces energy needs.

Measuring fluxes of metabolites in free-flying migrants has been acknowledged to be a major technical challenge [85]. As a surrogate, plasma metabolite concentrations have been used (e.g [16, 23, 28]). The metabolite concentrations used in this study, except GLU, have been shown to reflect turnover in studies on mammals and/or birds (see [16, 30]), although in some cases flux can vary without a change in concentration and vice versa [86]. The following discussion is based on the assumption that differences in plasma concentrations (except GLU) roughly reflect differences in substrate transport.

### Relationship between energy stores and fuel use

Long-term fasting inactive birds and mammals with high initial fat stores oxidize more fat than birds with low initial fat stores and thereby reduce the percentage of energy derived from protein [6], as also found in a within-species study in the wind-tunnel [87]. In our study, plasma metabolite levels indicate that this pattern of fuel supply can also be observed in free-flying migrating passerines. Plasma TG, GLYC and VLDL levels correlated positively with energy stores. Birds with high stores likely hydrolysed more fat and used the lipoprotein-based pathway to an increased extent. Surprisingly, FFA did not correlate positively but negatively with energy stores. One explanation is that the concentration of plasma FFA is limited because they must be transported bound to albumin. An increase of albumin in the blood, however, is limited because blood proteins (and therewith blood viscosity) have to be kept at a physiological level ([88]; see discussion in [9]). Therefore, FFA are withdrawn from circulation and resynthesized to TG. Accordingly, TG and VLDL levels increase and FFA levels decrease [9].

ßOHB levels did not correlate with energy stores. They are significantly increased in flying birds [16], but at high concentrations ßOHB reduces FFA release from adipose tissue [31, 89]. Hence, it seems that birds during migratory flight, whose success in migration critically depends on a very high proportional contribution of fat to total energy expenditure, balance their ßOHB synthesis at a level which does not impair fatty acid release from adipose tissue.

The negative correlation between energy reserves and UA in European robins supports the hypothesis that birds with less energy stores derive more energy from protein catabolism. This relationship, however, was not evident in the other two species, lending support to the hypothesis that protein breakdown is not directly related to rates of fat catabolism [20, 28]. GLU was not correlated with energy stores. GLU ranges within narrow limits and is of minor importance during endurance exercise and fasting.

### Changes of fuel supply during nocturnal migratory endurance flight and with flight behaviour

We found an increase in the plasma concentration of the metabolites of the fat metabolism during the first part of the night and, surprisingly, a decrease of all metabolites in the second half (except for FFA and GLYC). The increase at the beginning of the night can be explained by the birds turning on fat catabolism after the start of flight at dusk (see below). The decrease later during the night cannot be explained by concomitantly decreasing body energy stores, because these have been accounted for in the model. The most likely explanation is that this decrease is the consequence of a change in flight behaviour.

As shown by the atmospheric pressure data, night migrants over continental Europe prepare for landing already one or two hours ahead by slowly reducing flight altitude. The subsequent final landing may occur by a very steep descent or vertical fall during the last minutes (as observed by e.g. [90 and 91]). The decrease in the number of birds aloft after midnight (observed in many places, e.g [46, 47, 92, 93]) demonstrates that many nocturnal migrants land during darkness in the second half of the night. This is corroborated by the decrease of the mean flight height above ground and the negative vertical flight speed of small passerines after midnight. The slow decrease in flight altitude before landing might serve to look for a suitable resting habitat. Night migrants in the early morning are very rarely found in unsuitable habitats [94]. They are able to choose species-specific habitats even at night by visual and acoustic cues [94–96].

Early at night, nocturnal migrants at Col de Bretolet apparently are still in the phase of increasing fat catabolism from extramuscular fat deposits while UA, indicating protein catabolism, is already at high levels (see also [16]). An increase in the fat catabolites GLYC and FFA over 1–2 h after take-off has also been observed in various species in the wind tunnel and in homing pigeons [17, 18, 23–25]. However, an increase of TG and VLDL was not observed in these experimental studies, indicating that the experimental birds do not transport FA in the form of VLDL to the flight muscles. Direct measurements of protein and fat use of small passerines in the wind tunnel also show a shift of the relative contributions of protein and fat as a fuel towards more fat catabolism at the beginning of flight [2, 20, 28].

Garden warblers, and to a lesser degree pied flycatchers, reach higher plasma concentrations of all fat metabolites and have lower levels of UA and GLUK, hence probably rely more strongly on fat as a fuel, than European robins. Garden warblers and pied flycatchers are long-distance migrants wintering in sub-Saharan Africa, while European robins winter in southwestern Europe and north Africa. It is likely that the adaptations to a high reliance on fat as a fuel, and sparing protein, is more evolved in migrants flying long distances.

During prolonged flight, plasma metabolites of the fat and protein metabolism (FFA, GLYC, TG, ßOHB, UA) remain stable in red knots *Calidris canutus* flying up to 10 h and in yellow-rumped warblers *Setophaga coronata* flying up to 6 h in a wind tunnel [17, 28] or increase (FFA, ßOHB, UA) in homing pigeons flying up to 22 h [18, 25]. In contrast, the passerines in our study decreased the levels of most metabolites in the second half of the night (except for FFA and GLYZ, UA decreased only non-significantly). Because the metabolites of the lipid, protein and carbohydrate metabolism decreased, it is likely that reduced energy demands by flying downwards is the reason and not a shift in fuel composition. The sinking speeds measured by radar and atmospheric pressure loggers correspond to a reduction in energy demands of around 10–20% according to current flight models.

Exceptions are the FFA which remained virtually unchanged over night and GLYC which did not decrease after a long increase. An explanation for this might be that the reduction in lipolysis (to meet the reduced energy demands) is borne by a reduction of ßOHB as well as TG and VLDL, i.e. a reduction in the resynthesis of FFA and GLYC into TG, while FFA (with albumin as a carrier) and GLYC are released into the blood in similar amounts. The unchanged, relatively high FFA suppresses glycogen breakdown and retards the rate at which muscle glycogen is depleted [97]. Maintaining glycogen reserves might be crucial for survival after landing (see below).

Plasma GLU also decreased during the night in our study. In contrast, earlier studies showed either unchanged [17] or – after an initial decrease - increasing GLU levels with flight duration [25, 28]. The pattern observed in our study may reflect a metabolic adaptation in free-living nocturnal migrants. They will land in an unknown environment where they need burst flights to escape predators and hunt prey. Burst flights are fuelled with glycogen. Therefore, muscle glycogen reserves must be replenished before landing either directly by GLU or indirectly by metabolic by-products of GLU metabolism [98]. A replenishment of glycogen stores during fasting has been observed in several taxa ([99]; reviewed in [100]). The endogenous carbon sources needed for gluconeogenesis to replenish glycogen stores are likely GLYC and gluconeogenic amino acids [101–103]. Consistently, both GLYC and UA do not decrease before landing.

Remarkably, the three species used for measuring metabolite levels provided qualitatively very similar results. Also, the flight behaviour among the three species with geolocators was similar and corresponded to the general findings about flight behaviour of birds followed with radar. Nevertheless, the metabolism of the larger species (23–70 g) used for geolocators might differ from the smaller species used to measure metabolites (13–20 g), although we did not find an effect of body mass on metabolite concentrations during migratory flight across 30 passerine species (8–85 g) (unpubl. data). As well, habitat selectivity, and hence landing behaviour, of the three species with geolocators might be more specific than that of the three more widespread species used to measure metabolites. However, the radar data, which concern all species, provided similar information on flight behaviours. A study investigating flight behaviour and metabolism in the same species would be desirable.

## Conclusions

This study showed in free-flying nocturnal migrant passerines that fuelling endurance flight is dynamic. The composition of fuels (lipids versus protein) likely varies with the amount of body energy stores. The amount of energy needed varies with vertical speed. We suggest that a slow descent over about 1.5 h prior to the final landfall reduces energy demands to an extent which is noticeable in the plasma concentration of lipid, protein, and carbohydrate metabolites. The slow descent prior to landfall may prepare for the subsequent resting phase in two respects. One is the search for a suitable resting and refuelling habitat by visual and acoustic cues. The other might be that the reduced energy demand during descent may serve to refill glycogen stores. Both, an adequate refuelling habitat and the capability to perform burst flights to escape predators or to hunt prey are critical to continue migration.

### Electronic supplementary material

Below is the link to the electronic supplementary material.

Supplementary Material 1

## Data Availability

The datasets analysed during the current study are available at: Metabolite data at https://zenodo.org/records/10208834; radar observations at 10.5281/zenodo.10209093 and 10.5281/zenodo.10260473; northern wheatear tracks at https://zenodo.org/records/7471405; great reed-warbler tracks at https://zenodo.org/records/4017739; Eurasian hoopoe tracking data at 10.5281/zenodo.10260865.
