# Supplementary material for: Energy supply during nocturnal endurance flight of migrant birds: effect of energy stores and flight behaviour
Source: Mov Ecol. 2024 May 30;12:41. doi: 10.1186/s40462-024-00479-5 (PMC11140942; doi:10.1186/s40462-024-00479-5)

# Additional file

## Migration traffic rates measured by radar at Planachaux 2007

Figure 1. Mean migration traffic rate MTR (± SE) over the night for each hour (summed over all height intervals) during August, September, and October. Re-analysed data from S. Komenda-Zehnder, L. Jenni & F. Liechti (2010: Do bird captures reflect migration intensity? Trapping numbers on an Alpine pass compared with radar counts. Journal of Avian Biology 41: 434–44). Sample sizes of radar echoes were n = 14847 in August, n = 24822 in September, and n = 48984 in October.

The number of birds aloft at this study site in the Alps increases more slowly after dusk than at other sites (e.g. Aschwanden J, Schmidt M, Wichmann G, Stark H, Peter D, Steuri T, Liechti F. 2020: Barrier effects of mountain ranges for broad-front bird migration. Journal of Ornithology 161: 59–71). This is because fewer night migrants spend the day in the Alps than in the lowlands (about 50 km away) because of the lack of suitable habitats. Hence many birds need some time to reach our study site when starting at dusk and numbers build up more slowly.

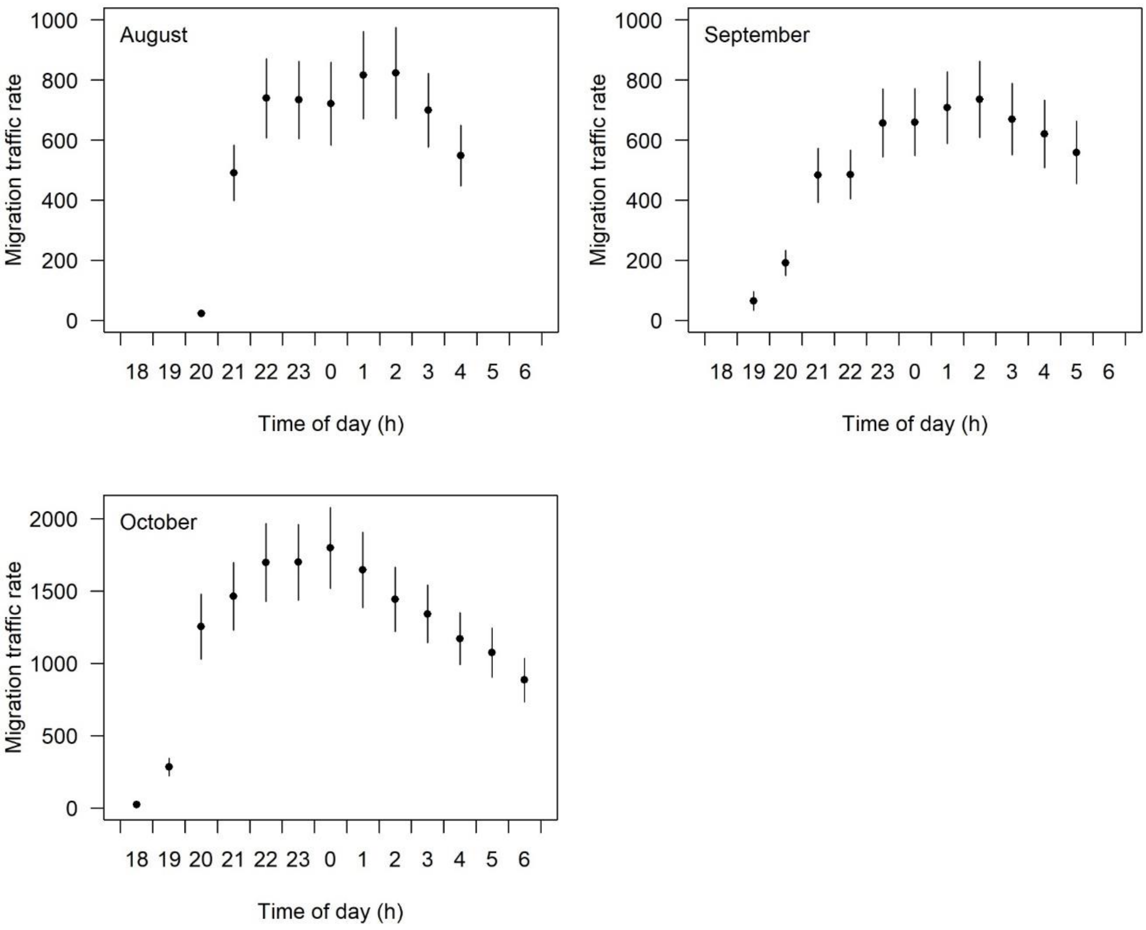

Supplement: Supplementary file 1 — Supplementary Material 1 [file 40462_2024_479_MOESM1_ESM.pdf]
